# Nutritional geometry and fitness consequences in *Drosophila suzukii,* the Spotted‐Wing Drosophila

**DOI:** 10.1002/ece3.3849

**Published:** 2018-02-11

**Authors:** Yvonne Young, Natasha Buckiewicz, Tristan A. F. Long

**Affiliations:** ^1^ Department of Biology Wilfrid Laurier University Waterloo ON Canada

**Keywords:** carbohydrates, diet, *Drosophila suzukii*, nutrition, nutritional geometry, protein, Spotted‐Wing Drosophila

## Abstract

Since its arrival to North America less than a decade ago, the invasive Spotted‐Wing Drosophila (*Drosophila suzukii*) has inflicted substantial economic losses on soft fruit agriculture due to its ability to oviposit into ripening fruits. More effective management approaches for this species are needed, but little is known about the factors that influence behavioral choices made by *D. suzukii* when selecting hosts, or the consequences that their offspring experience when developing in different environments. Using a nutritional geometry methodology, we found that the ratio of proteins‐to‐carbohydrates (P:C) present in media greatly influenced adult *D. suzukii* behavior and subsequent offspring development. Whereas adult flies showed a strong bias in their oviposition and association behaviors toward carbohydrate‐rich foods, larval survival and eclosion rate were strongly dependent on protein availability. Here, we explore the preference–performance hypothesis (PPH), in which females are predicted to oviposit on medias that provide the greatest offspring benefits, in regard to its relevance in *D. suzukii* behavior and consequences for management. Our results provide valuable insight into the ecology and evolution of this species that may hopefully lead to more effective management strategies.

## 1 INTRODUCTION

The Spotted‐Wing Drosophila, *Drosophila suzukii,* is an invasive agricultural pest known to attack a number of soft fruit species including blueberries, blackberries, strawberries, raspberries, and occasionally grapes (Bellamy, Sisterson, & Walse, 2013; Lee et al., 2011). Unlike most other drosophilids which seek out rotting fruit, *D. suzukii* exploits a different environmental niche, ripe or ripening fruit (Walsh et al., 2011). This is facilitated by the females’ large, serrated, ovipositor, which is used to cut the fruit's skin before laying eggs directly into the mesocarp (Atallah, Teixeira, Salazar, Zaragoza, & Artyom, 2014), ultimately leaving it unmarketable (Walsh et al., 2011). Since its initial arrival in North America, this species has resulted in yield losses of up to 80% (USDA NASS 2009) with Bolda, Goodhue, and Zalom (2010) estimating an annual economic loss of $500 million USD in California, Oregon, and Washington alone. To control and/or manage this species, it is imperative that we understand the factors that contribute to its success. Thus, information on host preference(s) and offspring performance is of great value since it is necessary for modeling population growth, monitoring spread, and designing better *D. suzukii* management plans.

To date*,* information on host preferences and the factors that influence the expression of these preferences in *D. suzukii* is very limited, and in those studies that have been conducted, the interpretation of the results is limited due to potentially confounding variables and/or nonrigorous methodologies. In a series of olfactory choice experiments, Abraham et al. (2015) and Bellamy et al. (2013) found that flies were differentially attracted to the volatile scents that originated from different fruit types, but this result does not necessarily translate into differences in oviposition rates. In the laboratory, simple (no‐choice or single‐choice) oviposition preference assays using whole fruit revealed that flies laid more eggs in raspberries than in blackberries, strawberries, or blueberries (Burrack, Fernandez, Spivey, & Kraus, 2013). In contrast, in Abraham et al. (2015)'s assays—using pureed fruit media—females laid more eggs in strawberry media than in all other choices. It is possible that these reported differences may be attributable to differences in the color, texture, scent, size, and/or shape of the options presented to the flies and may act as confounding variables that obscure host “preference.” Indeed, in an assay by Burrack et al. (2013) in which surface hardness in media was experimentally manipulated, egg‐laying rate among *D. suzukii* females decreased as surface hardness (and thus penetration force) increased. Thus, to understand the factors that shape behavioral decisions in *D. suzukii,* it is important to do so in a standardized fashion. Such is the approach we used to investigate the potential importance of proteins and carbohydrates to *D. suzukii* host preferences, an important first step toward understanding the ecology and life history of this invasive species.

Proteins and carbohydrates are two macronutrients that are most important for growth, reproduction, and survival in insects (Anderson, Kristensen, Loeschcke, Toft, & Mayntz, 2010; Carrel & Tanner, 2002; Jensen, McClure, Priest, & Hunt, 2015; Lihoreau, Poissonnier, Isabel, & Dussutour, 2016; Maklakov et al., 2008; May, Doroszuk, & Zwaan, 2015; Morimoto & Wigby, 2016; Raubenheimer & Simpson, 2003; Rodrigues et al., 2016). While dietary protein stimulates oogenesis and regulates vitellogenesis in females and stimulates the production of sperm in males (Jensen et al., 2015; Lee et al., 2008; Lihoreau et al., 2016; Maklakov et al., 2008; Pirk, Boodhoo, Human, & Nicolson, 2010; Reddiex, Gosden, Bonduriansky, & Chenoweth, 2013), carbohydrates are a source of energy for fat and glycogen synthesis (Friend, 1958). The fruit varieties potentially infested by *D. suzukii* vary considerably in both their ratio (P:C) and concentration (P + C) of proteins and carbohydrates, which may be underlying factor(s) influencing host preference, offspring survival, and egg‐to‐adult development in this species. For instance, raspberries, strawberries, and blueberries have P:C ratios of 1:5, 1:7, and 1:15 and P + C concentrations (g/kg) of 49, 56, and 108, respectively (Table S1). Understanding the relationship between an organism's performance and their nutritional acquisition is central to studies of “nutritional geometry,” a technique developed by Simpson. and Raubenheimer (1993) in which two variables (in our case, protein and carbohydrates) are experimentally manipulated across a “landscape.” Previous nutritional geometry studies show that both protein and carbohydrate availability is strongly associated with variation in foraging behavior, oviposition rate, development, reproduction, and longevity in a number of different insect species (e.g., Jensen et al., 2015; Lee et al., 2008; Lihoreau et al., 2016; Maklakov et al., 2008; Morimoto & Wigby, 2016; Reddiex et al., 2013; Rodrigues et al., 2016). For instance, while hissing cockroach females, *Gromphadorhina portentosa*, prefer to feed on relatively high‐protein foods (Carrel & Tanner, 2002), female *D. melanogaster* instead prefer foods with relatively higher carbohydrates, as sites for both their feeding and egg laying (Lihoreau et al., 2016; Rodrigues et al., 2016). A female's decision where to oviposit is also potentially important to the success of her offspring (e.g., Anderson et al., 2010; Morimoto & Wigby, 2016; Rodrigues et al., 2016) since the characteristics of the natal diet during development influence numerous fitness‐related traits. One such trait is adult body size, with larger males and females emerging from media of greater nutritional quality (e.g., Thomas, 1993). Larger individuals often exhibit higher fecundity (Kaspi, Mossinson, Kamensky, & Yuval, 2001; Lefranc & Bundgaard, 2000; Tantawy & Vetukhiv, 1960) and mating success (Partridge, Hoffman, & Jones, 1987; Partridge, Ewing, & Chandler, 1987). It has been proposed that selection will act on a species such that females will evolve a preference to oviposit in environments that will maximize their offspring's success (Gripenberg, Mayhew, Parnell, & Roslin, 2010). While this “preference–performance” (aka “Mother knows best”) hypothesis has found some empirical support (Bellamy et al., 2013; Hanks, Paine, & Miller, 1993; Rausher, 1979), there are several well‐documented cases in which females deposit eggs in suboptimal conditions indicating that this preference–performance relationship is not a ubiquitous phenomenon, even among closely related taxa (Konig, Wiklund, & Ehrlen, 2016; Lihoreau et al., 2016; Rausher, 1979). Preliminary evidence from studies using fruit suggests that *D. suzukii* might preferentially oviposit in fruit types that maximize offspring fitness, as females appear to lay more eggs in fruit type(s) that result in higher offspring survival (Bellamy et al., 2013; Burrack et al., 2013). However, standardized studies are needed to better test this hypothesis.

While *D. suzukii* are known to infest a number of different fruit species in North America, we know very little about what nutritional factors may influence these decisions and the consequences these decisions have to their offspring's future. While female *D. melanogaster* seek out medias that are relatively abundant in carbohydrates (Lihoreau et al., 2016; Rodrigues et al., 2016), the same may not necessarily be the case for *D. suzukii*, as the former lay their eggs in rotting fruit, which are colonized by protein‐rich molds and yeasts (Begon, 1986; Da Cunha, Dobzhansky, & Sololoff, 1951; Cooper, 1959), while the latter lay eggs in unspoiled fruits where the proteins may primarily come (initially) from the fruit itself, until such a time that microbes introduced by the female during oviposition, or as a result of her actions, have had time to flourish (Hamby, Hernandez, Boundy‐Mills, & Zalom, 2012; Mori et al., 2017). As a result, there may be considerable differences in the selection experienced by these two species, which has led to the evolution of different sets of adaptations. Jaramillo, Mehlferber, and Moore (2015) explored whether the shift from a protein‐rich to a protein‐poor nutritional niche had evolutionary consequences for *D. suzukii* life‐history traits, by comparing the performance of *D. suzukii* larvae grown on blueberries (P:C ~1:15) and on a standard laboratory fly media (P:C ~1:3). Overall, they found no major differences in survivorship, body size, rate of ovarian maturation, and early‐life fecundity. While these results suggest *D. suzukii* can develop on relatively little dietary protein, this study was potentially limited by the scope of P:C ratio diets tested, as well as by the fact that other differences besides P:C between the fly media and the blueberries could have confounded results. The most comprehensive study of *D. suzukii* utilizing a nutritional geometry framework has been that of Silva‐Soares, Nogueira‐Alves, Beldade, and Mirth (2017). Using a wide range of media that was manipulated in P:C and P + C to measure oviposition and offspring development, they found a strong bias toward egg laying in the lowest P:C medias and higher larval survival, faster development rates, larger adult masses, and greater ovariole numbers in individuals that were raised in environments that contained more protein. Nevertheless, much remains unknown about the nature of the relationship between adult oviposition behavior, larval diet, and development in this species. To understand the importance of P:C ratio to *D. suzukii* adult behaviors, we measured the activity and oviposition preference of flies among a relatively expansive range of P:C ratios by presenting flies with eight different artificial medias in “cafeteria”‐style arenas. Next, we tested the consistency of female behaviors by measuring egg‐laying preference in a no‐choice experiment using the eight different diets. Finally, we addressed the importance of the natal diet on development and adult traits by comparing the development (egg‐to‐adult survival, eclosion rates, and adult weights) of larvae under standardized competitive conditions on each of eight experimental diets. This information is potentially useful in furthering our understanding of evolutionary life‐history traits as well as for managing and/or mediating the effects of this invasive pest species.

## 2 MATERIALS & METHODS

### 2.1 Drosophila suzukii population history & culture protocols

All flies used in this experiment originate from a large (~1400 adults/generation) laboratory population of *D. suzukii*. This population was founded from a sample of individuals isolated from blackberries and raspberries collected from a Southern Ontario commercial farm during the summer of 2012 (described in Renkema, Wright, Buitenhuis, & Hallett, 2016) and which was shared with our laboratory in 2014 by Dr. Justin Renkema (University of Guelph). Since then, the flies have been cultured under standard laboratory conditions (25°C, 60% humidity, LD 12:12) on Rose's fly media (Rose, 1984). The population is cultured on a 21‐day cycle, whereby on day 1 of the cycle, flies are mixed *en masse* under light anesthesia (CO_2_) and transferred to a fresh set of vials containing ~10 ml of fly media, with 20–25 flies per vial. After 48 hr in these vials, the flies are transferred to a second set of fresh vials before being discarded 48 hr later.

### 2.2 Experimental diets

Preference and developmental performance of *D. suzukii* were analyzed using eight artificial diets in which the protein‐to‐carbohydrate ratio (P:C) was experimentally manipulated (P:C 1:12, 1:6, 1:3, 1:1, 2:1, 4:1, 8:1, 24:1). We chose these P:C ratios based on two criteria. First, these ratios allow us to compare our results with the work of other researchers that use similar nutritional geometry methods to measure *Drosophila* behavior and life history, specifically those studies by Lihoreau et al. (2016) and Rodrigues et al. (2016). Second, these values span the range of potential P:C ratios encountered by *D. suzukii* in their natural habitat. For instance, the ratios 1:12, 1:6, 1:3, and 1:1 span the P:C range commonly observed in farmed and wild fresh fruit commonly attacked by *D. suzukii* in North America (Table S1). The ratio 1:12 resembles the P:C ratio found in floral nectar (Kevan & Baker, 1983), a hypothesized energy source for adult *D. suzukii* (Tochen, Walton, & Lee, 2016). The ratios 2:1, 4:1, 8:1, and 24:1 represent the higher protein content potentially found in rotting fruits (Janzen, 1977; Matavelli, Carvalho, Martins, & Mirth, 2015; Silva‐Soares et al., 2017). While the protein and carbohydrate (P + C) concentrations in fruit vary from 40 to over 200 g/L (Table S1), we chose to focus on a single concentration of 70 g/L, because it represents the average of a large majority of soft fruit species attacked by *D. suzukii* (e.g., raspberries, blackberries and cherries; Table S1) and is similar to the P + C concentration of the standard media (64 g/L) we use to culture our laboratory population (Rose, 1984).

To generate the eight different medias, we manipulated the quantities of protein and carbohydrates (Table S2) in each recipe while keeping all other ingredients in the media constant, as outlined by Lihoreau et al. (2016). The ingredients used in our media were very similar to Lihoreau et al.'s media, with the exception that we used a 50:50 mix of light and dark corn syrup as the carbohydrate source, instead of sucrose. Corn syrup was used because it contains a 1:1 ratio of fructose and glucose similar to that of fruit; it is the main carbohydrate source we use to culture our laboratory population and (unlike sucrose) has not been linked to a decrease in female fecundity and lifespan in *Drosophila* (Begon, 1986; Hassett, 1948; Lushchak et al., 2013). We used a 50:50 mix of whey (GNC #386306) and casein (Sigma‐Adrich, C3400) for the protein. All media includes Vanderzant vitamin mixture (Sigma‐Aldrich, V1007; 0.25 g/L), methylparaben (Bioshop, HYD202; 4 g/L), and propionic acid (Fisher Scientific A258‐500; 1.5 g/L). In all cases, we added commercially available flaked nutritional yeast (10 g/L) (Bulk Barn, 000939), a common ingredient in the media of similar experiments with *Drosophila* (Lihoreau et al., 2016; Rodrigues et al., 2016). As such, the protein (0.46 g/g) and carbohydrate (0.38 g/g) content provided by the yeast was incorporated into the calculations. All media contained 2% agar (Bio Basic Canada Inc. FB0012) and was dyed with green food coloring (Club House Brand) for greater background contrast during egg counting.

### 2.3 Assay 1: fly movement and oviposition in a “cafeteria” choice environment

We first set out to quantify the behavior of *D. suzukii* in an environment where they have access to a wide range of P:C media types. We did so by first collecting 160 sets of 15 adult male and 15 female flies from our stock population. These flies were collected on days 18–24 of their culture cycle and were fully mature and likely nonvirgin. Each set of flies was placed, under light anesthesia (CO_2_), into a single vial containing 10 ml of lightly yeasted culture media and stored in an incubator for 48 hr prior to the start of the assay.

The “cafeteria‐style” choice arenas (Figure S1) we used to measure fly behavior consisted of transparent plastic boxes (KIS Omni Box, 20.3 × 15.9 × 9.6 cm) to which we added mesh‐covered vent holes along the upper edges. At the bottom of each chamber, we arranged eight petri dishes (BD Falcon, 31 mm) that each contained 8 ml of a different P:C media. We placed the dishes in the arenas ~2 hr before the introduction of the flies. The arenas, 80 in total, were housed in a well‐lit and quiet room.

The assay began when we transferred (without anesthesia) two vials of flies (60 flies in total) into each of the arenas. The flies were then left in the chambers for 25 hr, with a survey of fly locations made at 1, 4, 8, 21, 23, and 25 hr post‐introduction. This time range is meant to capture a wide “view” of the potentially variable periods of *D. suzukii* activity levels, as activity levels in this species are known to vary significantly depending on the time of day (Ferguson, O'Neill, Audsley, & Isaac, 2015). During each survey, the number and sex of all the flies located on the media surface of each petri dish were recorded. At the end of the 25‐hr period, all of the flies were removed from the arenas and the eggs laid on the surface of each of the media in the petri dishes were immediately counted.

### 2.4 Assay 2: oviposition in a no‐choice scenario

In order to investigate egg‐laying behavior in a “no‐choice” environment, we collected 880 females from our laboratory population. These flies were collected on days 18–24 of their culture cycle, were fully mature, and presumably mated. Each female was placed, individually, into a vial containing 2 ml of one of the 8 P:C medias described above. Vials with flies were incubated for 36 hr before all females were removed and the number of eggs laid in each vial was counted.

### 2.5 Assay 3: larval development on the eight different P:C diets

We next set out to quantify the development of *D. suzukii* larvae on media with different P:C ratios (but standardized initial levels of larval competition). The assay (which shall hereafter be referred to as the “T+ assay”) began by collecting eggs laid by adult flies from our laboratory population. This was done by placing flies into half‐pint laying chambers outfitted with 35‐mm petri dish lids (BD Falcon) containing a grape‐agar media (Sullivan, Ashburner, & Hawley, 2000) for ~18 hr. Eggs were sorted into groups of 20 and transferred into vials containing 10 ml of one of the 8 P:C media types, replicated 50 times per treatment. These vials were incubated, and starting 12 days later, all eclosed adult flies were removed, sexed, and counted every 48 hr, a schedule that continued until day 22. The first 50 females and the first 50 males collected from each media treatment on the census days were immediately frozen for later weighing. Flies were weighed by first placing them into a drying oven set at 70°C overnight and weighed on a Sartorius ultramicrobalance to the closest 0.1 μg.

Later, to investigate the possibility that *D. suzukii* larvae might benefit from the protein originating from microbial growth in the media, as is seen in other *Drosophila* species (Begon, 1986; Da Cunha et al., 1951; Cooper, 1959; Lihoreau et al., 2016), we conducted a follow‐up experiment (hereafter referred to as the “T− assay”). In this assay, we omitted the addition of antimicrobials (Tegosept and propionic acid) in the media, but otherwise followed the same experimental protocols used in the first developmental assay, except with fewer replicates (25) per treatment. Flies that eclosed as adults were removed, sexed, and counted every 24 hr for a total of 22 days.

### 2.6 Statistical analysis

We used R 3.3.1 (R Core Team 2016) for all statistical analyses. The location of male and female flies on the eight different P:C medias was analyzed both together and separately by sex using general linear models (GLMs) constructed with quasibinomial error distributions. In each model, the sum of all counts of flies on the surface of the petri dishes containing media over the course of the 25‐hr observation period was the dependent variable and the total count of flies on all petri dishes in the chamber throughout the assay was the binomial denominator. The significance of treatment was determined using the *Anova* function (in the *car* package, Fox & Weisberg (2011)), with type II sums of squares. To determine the differences in egg‐laying behavior associated with different media types, we constructed a GLM with a quasipoisson error distribution. A model was created for each class of behavioral or fitness response, with treatment as an independent factor. The significance of treatment was determined using the *Anova* function, and specific differences in the number of eggs laid on each media type were determined using a Tukey's HSD test. Egg‐laying activity in the no‐choice scenario was also analyzed using a GLM with quasipoisson error distribution. To see if females exhibited a similar preference for egg‐laying site when given no choice versus a choice in media, we performed a Spearman correlation test in which we examined the number of eggs laid on each type of media where flies were given a choice and no choice. Survivorship among the different treatments was analyzed by fitting a GLM with a quasibinomial logit to the number of flies that eclosed in each vial in each treatment as the dependent variable, and the initial number of eggs added to the vial was the binomial denominator. In order to measure potential differences in eclosion rate in different media, we performed a Kruskal–Wallis (rank‐sum) test on the number of flies that eclosed each day followed with a *post hoc* comparisons of medians using the *kruskal.mc* function in the *pgirmess* package (Giraudoux., 2016). Finally, the normally distributed male and female fly weights were analyzed separately by sex using a one‐way ANOVA followed by *post hoc* Tukey's HSD test to determine where the differences in adult weight lay between media treatments.

## 3 RESULTS

### 3.1 Fly distribution & oviposition in “choice” chambers

**Figure 1:**
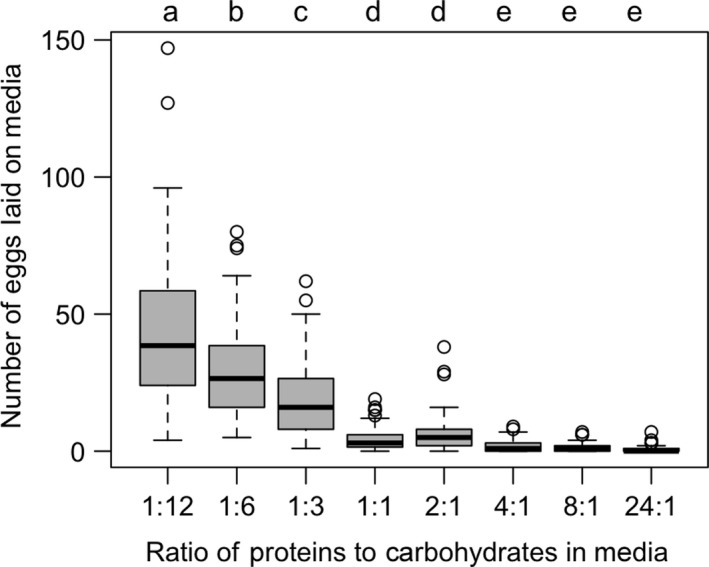
Oviposition preference among medias differing in protein: carbohydrate (P:C) ratio in choice environments. Boxplots of numbers of eggs female *Drosophila suzukii* laid on each of the eight different P:C medias in each choice chamber (80 chambers in total) over a 25‐hr period. The box encloses values between the first and third quartiles of the data (the interquartile range (IQR)), whereas the horizontal bar within the box indicates the median. Whiskers extend from the box to largest/smallest values that are within 1.5 ×  the IQR of the box. Values outside that range are outliers and are indicated by circles. Boxplots that are not sharing a letter have significantly different means

Adult *D. suzukii* flies distributed themselves nonrandomly among the eight P:C media in the choice arenas, with the greatest number of flies associating on the highest carbohydrate (1:12) media over the 25 hr of observation. This pattern was seen in both sexes when they were analyzed separately by sex (GLM: females: LLR χ^2^ = 350.05, *df *= 7, *p *<* *.001; males: LLR χ^2^ = 610.18, *df* = 7, *p *<* *.001) and when pooled together (LLR χ^2^ = 750.11, *df* = 7, *p *<* *.001; Figure S2). Similarly, the number of eggs laid on the media differed significantly between media types (GLM, LLR,χ^2^ = 1458.9, *df* = 7, <0.001). The greatest number of eggs was laid in the media with the highest carbohydrate‐to‐protein ratio, 1:12 (mean = 42.7 eggs or ≈40% of eggs laid/chamber), and progressively fewer eggs were laid on media with a decreasing carbohydrate‐to‐protein ratio (Figure 1). The strong relationship between oviposition rate and macronutrient composition were also seen when our experimental media ratios were treated as continuous variables (Figures S3 and S4).

### 3.2 Oviposition in “no‐choice” vials

The mean number of eggs laid by single *D. suzukii* in the “no‐choice” vials differed between the media treatments (GLM, LLR, χ^2^ = 45.64, *df* = 7, <0.001). We saw greater oviposition on those media with low P:C ratios (such as 1:12 and 1:6) than on those with high ratios (i.e., 24:1). We observed a significant positive correlation between the number of eggs laid on the media types in the no‐choice and choice experiments (Spearman's Rho = 0.785, *S* = 18, *df* = 7, *p *=* *.028).

### 3.3 Development & survivorship

**Figure 2:**
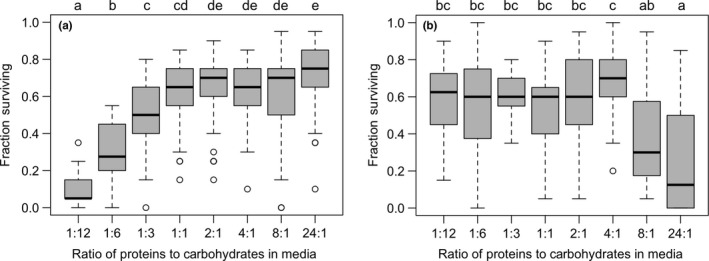
Larval survivorship on medias differing in protein: carbohydrate (P:C) ratio. Boxplots of proportion of flies that eclosed as adults within 22 days following the transfer of a standardized number of eggs (20) into vials containing artificial media a) with antimicrobials and b) without antimicrobials. The box encloses values between the first and third quartiles of the data (the interquartile range [IQR]), whereas the horizontal bar within the box indicates the median. Whiskers extend from the box to largest/smallest values that are within 1.5 ×  the IQR of the box. Values outside that range are outliers and are indicated by circles. Boxplots that are not sharing a letter have significantly different means

The egg‐to‐adult survivorship of *D. suzukii* differed depending on the type of media that the larvae developed on, albeit with opposing trends between the two experiments. In media where antimicrobials had been added to the media (T+ assay; GLM: LLR χ^2^ = 557.15, *df* = 7, *p *<* *.001), the greatest mortality arose on carbohydrate‐rich medias and the greatest survivorship on protein‐rich medias (Figure 2a), whereas in the media in which antimicrobials were omitted (T− assay; GLM: LLR χ^2^ = 46.54, *df* = 7, *p *<* *.001), the greatest mortality arose on protein‐rich medias and the greatest survivorship on carbohydrate‐rich medias (Figure 2b). The strong relationship between survivorship and macronutrient composition was also seen when our experimental media ratios were treated as continuous variables in both our assays that included and excluded antimicrobials (Figure S5). The number of males and females that eclosed did not differ between the eight media types, indicating there was no diet‐related sex‐biased survivorship (T+ assay: GLM: LLR χ^2^ = 4.8397, *df* = 7, *p *=* *.680; T− assay: GLM: LLR χ^2^ = 8.8596, *df* = 7, *p *=* *.2629). Overall, we saw the flies’ development speed depended on the type of experience in both experiments (Kruskal–Wallis test: T+ assay: females: χ^2^ = 132.8, *df*  = 7, *p *<* *.0001; males: χ^2^ = 173.73, *df* = 7, *p *<* *.001; Kruskal–Wallis test: T− assay: females: χ^2^ = 157.82, *df* = 7, *p *<* *.0001; males: χ^2^ = 228.9, *df* = 7, *p *<* *.001; Figure S6). In media that contained antimicrobials, flies developing on carbohydrate‐rich media tended to eclose later than those on protein‐rich media (Figure S6a,b), whereas in the media that did not contain antimicrobials, the opposite pattern was seen (Figure S6c,d). Flies developing on different media (with antimicrobials) also eclosed at different mean masses (ANOVA: males: *F*_7,340_ = 5.534, *p *<* *.001; females: *F*_7,378_ = 3.227, *p *<* *.001). Pairwise comparisons of weights do not suggest any specific directional pattern, except perhaps a tendency for flies in the extreme ratios eclosing at a lighter weight than others (Figure S7).

**Figure 3:**
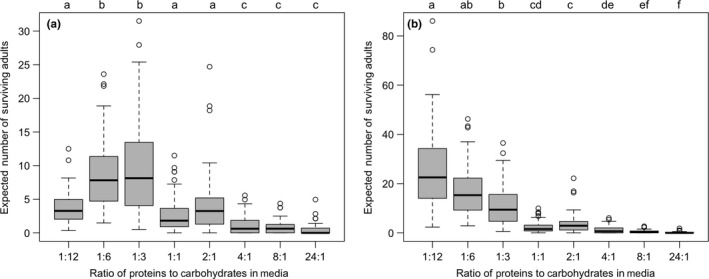
Predicted number of surviving adults on medias differing in protein: carbohydrate (P:C) ratio. Boxplot of number of adults predicted to eclose on substrates of differing P:C ratios based on oviposition rates in a choice environment and survivorship values derived from the assays in media, a) inhibited microbial growth and b) supported microbial growth. In both analyses, there were significant differences in the number of expected offspring eclosing from each media type (Kruskal–Wallis tests: a: χ^2^ = 356.01, *df* = 7, *p *<* *.0001; b: χ^2^ = 476.45, *df* = 7, *p *<* *.001). The box encloses values between the first and third quartiles of the data (the interquartile range [IQR]), whereas the horizontal bar within the box indicates the median. Whiskers extend from the box to largest/smallest values that are within 1.5 ×  the IQR of the box. Values outside that range are outliers and are indicated by circles. Boxplots that are not sharing a letter have significantly different medians

## 4 DISCUSSION

The Spotted‐Wing Drosophila, *D. suzukii*, is an invasive species responsible for significant economic damage to agriculture ever since its arrival to North America less than a decade ago (Bolda et al., 2010). However, there has been surprisingly little research conducted on host preference behaviors or on the fitness consequences associated with the choice of oviposition host in this species. Here, we use a series of assays based on a nutritional geometry framework to explore host‐associated preference(s) in adult *D. suzukii* and test the suitability of different developmental environments to their offspring. We found a strong oviposition preference for media that contained a relatively low protein: carbohydrate ratio and a strong variation in larval survival and development across the P:C ratio spectrum that was in direct relation to the presence or absence of antimicrobials in the media. Whereas larval survival and development were found to be greatest on media with high P:C ratios when antimicrobials were present, the opposite was found to be true when antimicrobials were absent. We explore the potential causes and consequences of these conflicting results from evolutionary, ecological, and management perspectives.

In our first set of experiments, we observed the behaviors of adult flies of both sexes in “cafeteria‐style” arenas, where flies were able to freely visit eight different medias that spanned a wide range of P:C ratios. We observed a significant nonrandom pattern in both the physical location of males and females, as well as in the number of eggs that were laid over the 25‐hr observation period. Consistent with observations made by Lihoreau et al. (2016) on *D. melanogaster*, media with lower P:C ratios were consistently visited more frequently than those media with high P:C ratios (Figure S1). A reason that flies exhibit this seeming preference may be due to the numerous metabolic benefits associated with this macronutrient (Maklakov et al., 2008). As is seen in other insects, the success of adult *D. suzukii* may depend on one's ability to perform energy‐demanding activities (Maklakov et al., 2008), such as rigorous courtship displays (Revadi et al., 2015) and daily foraging for mates, nutritional resources, and hosts. Carbohydrates, being a rapidly metabolized form of energy, may be preferred by *D. suzukii* for meeting their energy demands and/or optimizing performance. These high‐carbohydrate medias also were the site of much greater levels of oviposition, compared to media with higher P:C ratios (Figure 1). Similarly, when females were placed into “no‐choice” vials, those in the vials with low P:C media laid more eggs than those in the vials with high P:C media. Together, these results independently indicate a strong ovipositional preference for medias that are rich in carbohydrates over those that are rich in proteins. Our results are consistent with recent nutritional geometry studies by Rodrigues et al. (2016), Lihoreau et al. (2016), and Schwartz, Zhong, Bellemer, and Tracey (2012) that each found female *D. melanogaster* to lay a greater number of eggs on low P:C ratio media. Further studies on macronutrient requirements in this species may provide insight into the behaviors and dietary needs of *D. suzukii*, advancing our understanding of evolutionary processes, as well as furthering the management of this species.

Our oviposition results exhibit a similar pattern to that observed by Silva‐Soares et al. (2017). In their study, females had the choice of three different isocaloric media (spanning the range of P:C ratios of 1:1 to 1:8) and saw the greatest egg laying (measured over a 6‐h period) in the lowest P:C environment. Our results differ, however, from the study by Burrack et al. (2013) in which flies were given a “single choice” between raspberries (P:C ratio of ~1:4) and blueberries (P:C ratio of ~1:15) and in which more eggs were laid on the former than on the latter. The reason for these differences may potentially be explained by differences in the experimental design of our two studies. Whereas we presented females with an artificial media for oviposition, Burrack et al. used intact blackberry, strawberry, blueberry, and raspberry fruits. These various fruits differ in color, aroma, size, texture, the force necessary to penetrate the skin (e.g., blueberries require 3.4 times more force than raspberries; Burrack et al., 2013), and total macronutrient concentration (the P + C content of blueberry is approximately twofold higher raspberries at ~108 g/kg of fruit), any of which may have influenced their results. Our use of a standardized media potentially controlled for many of these confounding variables, and thus, we were able to reveal an underlying *D. suzukii* preference.

As *D. suzukii* larvae have a limited capacity for dispersal, a female's decision where to lay her eggs will dictate what kind of developmental environment that her offspring experience. Given the strong bias toward greater oviposition on media with low P:C ratios, our development assays allowed us to test whether or not females choose to oviposit in environments that result in the highest offspring performance, as predicted by the preference–performance hypothesis (Gripenberg et al.,^2010^). In our initial developmental assay (T+), in which media (prepared in the same manner as Lihoreau et al., 2016) contained antimicrobial agents (methylparaben and propionic acid), we observed the greatest survivorship and fastest development on the media with the highest P:C ratios. In our follow‐up (T−) assay (in which mold inhibitors were not added to the media), we saw the opposite pattern of larval development, with the greatest success associated with the media with the lower P:C ratios. While initially seeming at odds with each other, due to their opposite patterns of development, together the T+ and T− assays in fact provide complementary information on the supreme importance of protein to the egg‐to‐adult survival and proper development of *D. suzukii*, and the sources of said protein in nature. In our T+ assay, we suppressed the growth (of some) of the bacteria and fungi (potentially inoculated by females during oviposition; Mori et al., 2017) that would otherwise proliferate in our media. As these microbes (especially yeasts) are often the primary source of nutrients for many *Drosophila* sp. (e.g., Starmer, 1981; Broderick & Lemaitre, 2012; Hamby et al., 2012), the greatest offspring development was in the media whose recipe contained the most protein. When we did not incorporate the antimicrobial agents in the T− assay, larvae did well in those environments, that is, the high carbohydrate media, in which fungi and bacteria flourished (Tournas & Katsoudas, 2005). The effect of the microbial decay of fruits results in the rapid decrease in sugars and the corresponding increase in proteins (Silva‐Soares et al., 2017), a pattern that was potentially inhibited in the T+ assay, but could proceed in the T− assay. These results complement the recent findings of Hardin, Kraus, and Burrack (2015), Bellutti et al. (2017), and Silva‐Soares et al. (2017), who all found that larval performance in *D. suzukii* is greater in those environments in which protein is more readily available. The contribution of protein associated with microbial decay may also help explain Lee et al.'s (2011) observation that development of *D. suzukii* larvae was higher in fruit cultivars with greater brix levels (% sugar content). In Jaramillo et al.(2015) study, no effect of P:C ratio was observed to effect the egg‐to‐adult survivorship of larvae developing. However, that study was limited to only two different environments, one of which contained methylparaben and propionic acid, which limits direct comparisons to other studies. Overall, our results suggest that *D. suzukii* female oviposition site preferences align with the developmental environment that would provide their offspring with the best nutritional resources (as predicted by the preference–performance hypothesis, Gripenberg et al., 2010). This may also be true in species of *Drosophila* that are unable to parasitize ripe fruit (and instead lay their eggs in rotting fruits) that also exhibits a strong bias toward ovipositing in environments high in carbohydrates (*D. melanogaster*, Lihoreau et al., 2016; *D. biarmipes,* Silva‐Soares et al., 2017) and in which protein availability is strongly associated with their larval development and survival.

One potential downside of the strong bias toward high‐carbohydrate oviposition sites in *D. suzukii* is that their offspring are likely to encounter greater levels of intraspecific competition (and/or greater exposure to waste products) during their larval phase, which could mediate growth rates (reviewed by Ashburner et al., 2005). The negative effects of larval crowding on *D. suzukii* in a study by Hardin et al. (2015) were manifested starting at ~20 larvae/13 g media (Hardin et al., 2015), which is comparable to our developmental assay densities (initially 20 eggs/~10 g media). This may explain our observed pattern of adult masses in our T+ assay: In the low P:C treatments, fly weight may have been negatively affected by the poor nutritional environment (also seen in their high mortality rates), while in the high P:C treatments, the higher survival rates ultimately may have resulted in stronger factors adversely affecting adult weights. Eggs developing in media of intermediate P:C ratios, then, potentially avoid the worst of these two effects and are thus able to develop into larger adults.

Our analyses are not without some potential caveats. First, the artificial diets used in our assay obviously differ from the types of oviposition and nutritional environments that *D. suzukii* encounter in nature in many different ways (e.g., scent, texture, color, taste, surface hardness, size, spacing, orientation) which could have influenced the perception of the quality of the environment by the ovipositing females and that may have biased our observations. Furthermore, the specific ingredients chosen for our recipes may have had unanticipated effects on larval development, either directly (from consumption) or indirectly (by influencing the abundance and/or diversity of microbes that grew in our vials, especially in our T− assay). These are valid concerns, which should not be taken lightly, but are inevitable in any study attempting to examine specific components of complex subjects such as behavior and development. Future studies, building off our results, should attempt to incorporate more complexity and realism into their designs in an attempt to bridge the gap between the laboratory and nature.

*Drosophila suzukii* is a serious economic pest, and more effective control strategies are urgently needed. As *D. suzukii* appears to be strongly attracted to carbohydrate‐rich media, this preference may be exploited in attract and kill strategies such as baited trapping. In field experiments conducted by Iglesias, Nyoike, and Liburd (2014), evidence of this potential application can be seen when the addition of sugar to wine and apple cider vinegar baits resulted in greater *D. suzukii* captures. In addition, as greater quantities of carbohydrates are associated with improved survivorship of juvenile offspring in the T− assay, then all else being equal (e.g., fruit size, P:C, color, micronutrients), farmers may benefit by focusing management efforts (i.e.*,* spraying and trapping) on more sugary fruits, which may be acting as an important source of population recruitment. This strategy may be particularly useful in fields comprised of different cultivars of the same fruit type that differ in their carbohydrate composition.

Nutritional geometry is an effective and well‐established framework that is highly suitable for the investigation of targeted questions related to nutrition. By reducing the number of variables, the complexity of outcomes that accompany a complete diet is controlled, allowing us to see how each nutritional variable affects life‐history traits, as well as to see how these variables interact. Our results highlight the importance that P:C ratios have on adult behavior and larvae performance in *D. suzukii*. To gain a more comprehensive understanding of the nutritional ecology of *D. suzukii*, further studies are needed to incorporate the potential interactions between the nutritional composition of fruits and microbial growth. Furthermore, the impact of different microbial species on *D. suzukii* development will need to be assessed thoroughly. Understanding these factors will be an important step toward developing innovative management techniques that are based on the insects’ own species‐specific biology.

## ACKNOWLEDGMENTS

This work was supported by a Natural Sciences and Engineering Council of Canada (NSERC) Discovery Grant held by TAFL. We would like to thank the members of the Long Lab for their assistance in “fly‐pushing.” Drs. Scott Ramsay, Jennifer Baltzer, and Jonathan Newman, as well as two anonymous reviewers, are thanked for their constructive suggestions that improved the quality of this manuscript. This research was conducted on the traditional territory of the Neutral, Anishnawbe, and Haudenosaunee peoples. The authors have no conflicts of interest to declare.

## CONFLICT OF INTEREST

None declared.

## AUTHOR CONTRIBUTIONS

YY and TAFL conceived and designed the experiments. YY, NB, and TAFL performed the experiments. YY and TAFL analyzed the data. YY, NB, and TAFL wrote the manuscript.
