# Supplementary material for: Nutritional geometry and fitness consequences in Drosophila suzukii, the Spotted‐Wing Drosophila
Source: Ecol Evol. 2018 Feb 11;8(5):2842–51. doi: 10.1002/ece3.3849 (PMC5838031; doi:10.1002/ece3.3849)
Supplement: Supplementary file 1 [file ECE3-8-2842-s001.pdf]

Supplemental Table S1. The protein to carbohydrate ratios and concentrations of fruit species attacked by *D. suzukii* in North America. Nutritional information accessed from the USDA Nutrient Database (https://ndb.nal.usda.gov/ndb/).

Species	Protein: Carbohydrate	Protein + Carbohydrate (g/kg)
Blueberry (*Vaccinium spp*.)	1:15	108
Plum (*Prunus spp*.)	1:14	107
Elderberry (*Sambucus spp*.)	1:10	77
Peach (*Prunus persica*)	1:9	89
Sweet Cherry (*Prunus spp*.)	1:8	90
Nectarine (*Prunus persica*)	1:7	91
Sour Cherry (*Prunus spp*.)	1:7	96
Strawberry (*Fragaria spp*.)	1:7	56
Apricot (*Prunus spp*.)	1:6	104
Mulberry (*Morus spp*.)	1:6	94
Currant (*Ribes spp*.)	1:5	84
Gooseberry (*Ribes spp*.)	1:5	52
Raspberry (*Rubus spp*.)	1:5	49
Chokecherry (*Prunus virginiana*)	1:5	169
Blackberry (*Rubus spp*.)	1:4	63
Grape (*Vitis spp*.)	1:3	220

Supplemental Table S2. Exact quantities of protein (P) and carbohydrates (C) in each 1 L ratio recipe.

Ratio P:C	1:12	1:6	1:3	1:1	2:1	4:1	8:1	24:1
Protein (g/L)	5	10	18	35	47	56	62	67
Carbohydrates (g/L)	65	60	53	35	23	14	8	3

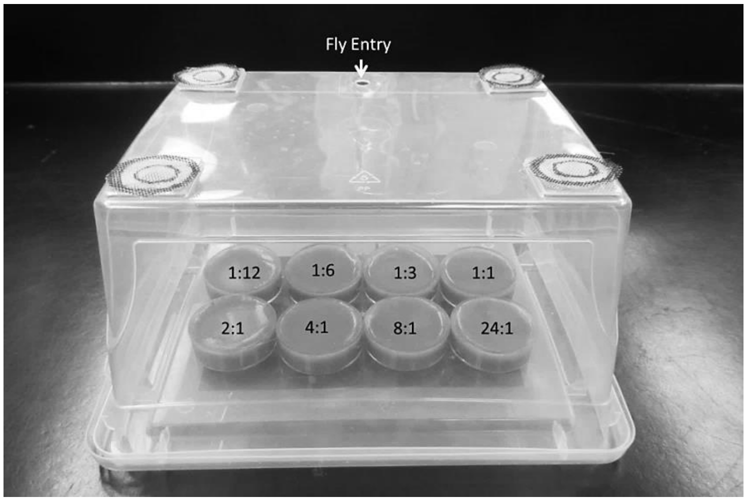

Supplementary Figure S1 Picture of a “choice” chamber containing dishes of the eight different protein-to-carbohydrate (P:C) ratio medias.

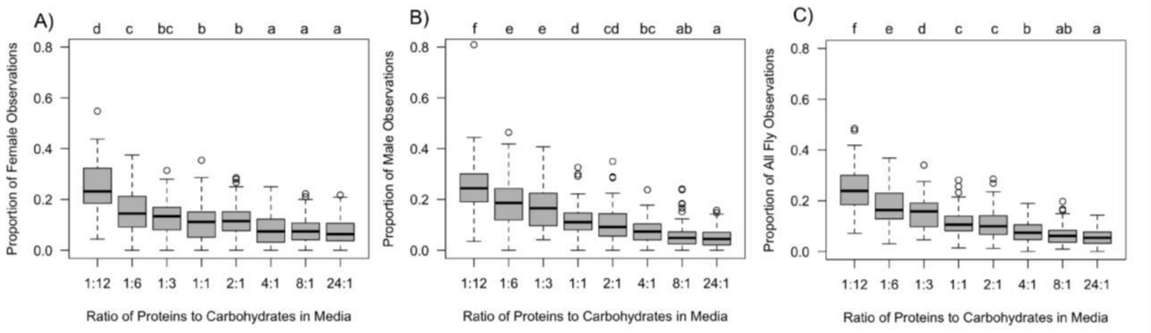

Supplementary Figure S2 Boxplots of distribution of flies among medias differing in protein to carbohydrate ratio (P:C). Proportions of time *Drosophila suzukii* females (A), males (B) and both sexes together (C) spent on each of the eight different P:C medias in the choice chambers at any one time over a 26-hour observation period. The box encloses values between the first and third quartiles of the data (the inter-quartile range (IQR)), whereas the horizontal bar within the box indicates the median. Whiskers extend from the box to largest/smallest values that are within 1.5 × the IQR of the box. Values outside that range are outliers and are indicated by circles. Boxplots that are not sharing a letter have significantly different means.

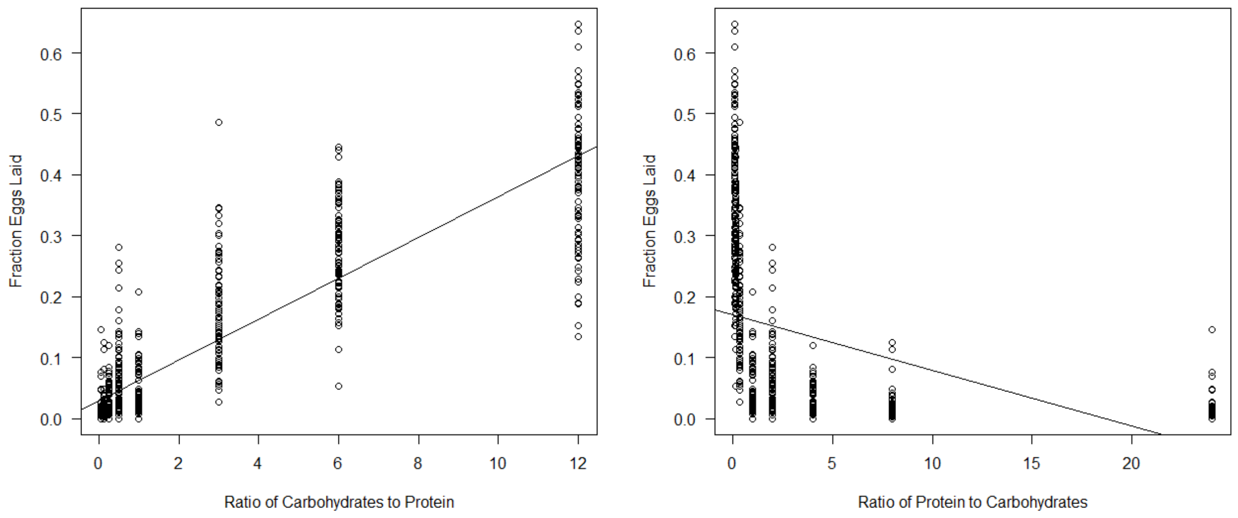

Supplementary Figure S3. Scatter plots illustrating the regression lines between the total number of eggs laid in the 'choice' scenario in relationship to A) the carbohydrate to protein ratio in the media (slope of 0.033 and y-intercept of 0.029) and B) the protein to carbohydrate ratio in the media (slope of -0.009 and y-intercept of 0.169). ANOVA $F=2289.5$, $df=1, 638$, $p<2.2\times10^{-16}$

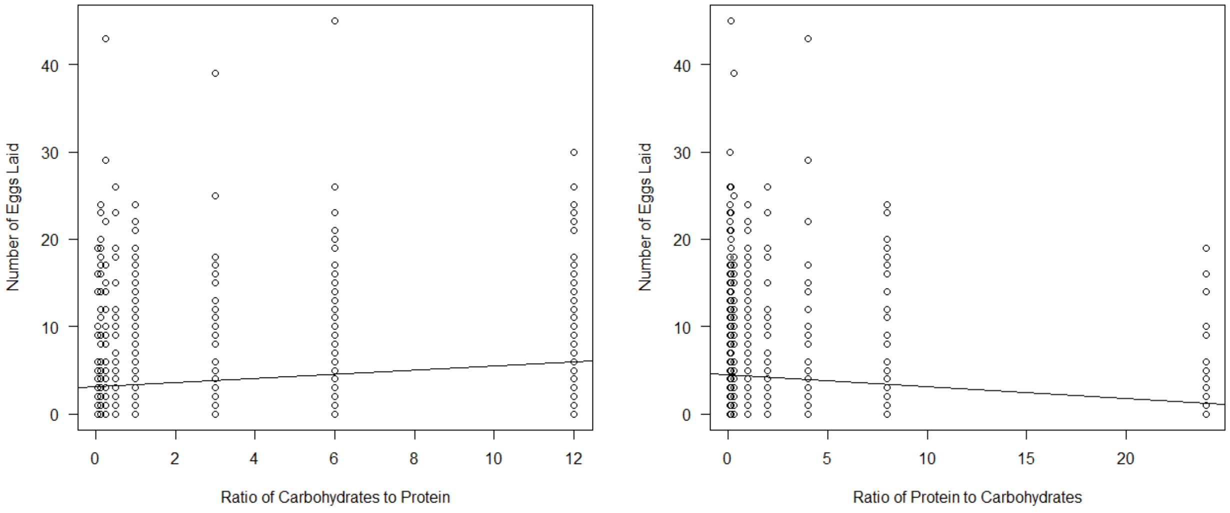

Supplementary Figure S4. Scatter plots illustrating the regression lines between the total number of eggs laid in the 'no choice' scenario in relationship to A) the carbohydrate to protein ratio in the media (slope of 0.238 and y-intercept of 3.095) and B) the protein to carbohydrate ratio in the media (slope of -0.013 and y-intercept of 4.45). ANOVA $F=175.37$, $df=1, 638$, $p<2.2\times10^{-16}$

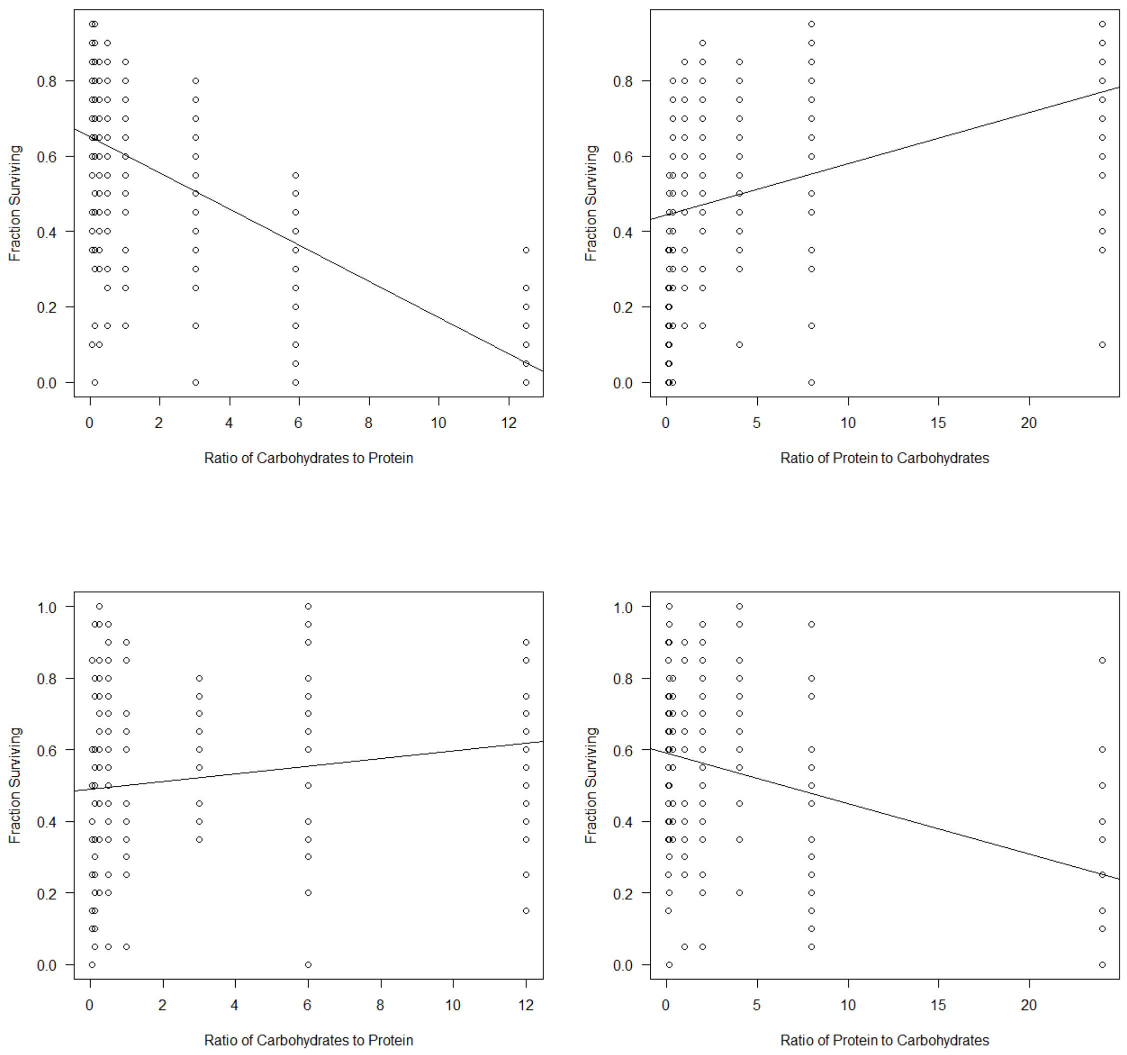

Supplementary Figure S5. Scatter plots illustrating the regression lines between the proportion of offspring surviving in relationship to A) the carbohydrate to protein ratio in the media with ***antimicrobials added,* T+** (slope of -0.048 and y-intercept of 0.652; ANOVA F=539.61, df=1, 396, p<2.2x10^{-16}; ANOVA F=76.524, df=1, 396, p<2.2x10^{-16}), B) the protein to carbohydrate ratio in the media with ***antimicrobials added* T+** (slope of 0.44 and y-intercept of 0.014; ANOVA F=5.065, df=1, 172, p=0.026; ANOVA F=36.795, df=1, 172, p<8.1x10^{-9}), C) the carbohydrate to protein ratio in the media with ***antimicrobials omitted* T-** (slope of 0.490 and y-intercept of 0.011), and D) the protein to carbohydrate ratio in the media with ***antimicrobials omitted* T-** (slope of -0.014 and y-intercept of 0.591).

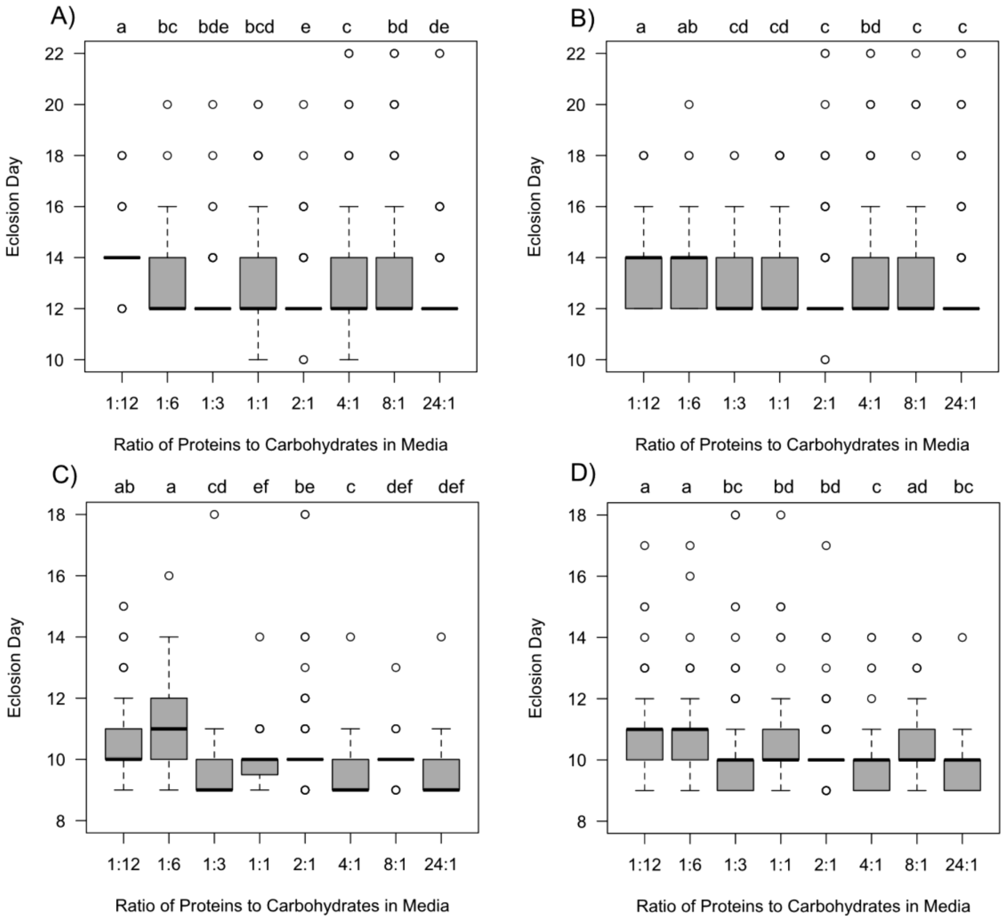

Supplementary Figure S6. Boxplots of dates of eclosion from pupae of adult *Drosophila suzukii* on medias differing in their protein: carbohydrate (P:C) ratio. Left panels (A & C) indicate observations made for males, while right panels (B &D) indicate observations made for females. Top panels (A &B) represent data collected from the assay in which antimicrobials were added to media while bottom panels (C &D) represent data collected from the assay in which antimicrobials were omitted from the media. The box encloses values between the first and third quartiles of the data (the inter-quartile range (IQR)), whereas the horizontal bar within the box indicates the median. Whiskers extend from the box to largest/smallest values that are within 1.5 × the IQR of the box. Values outside that range are outliers and are indicated by circles. Boxplots that are not sharing a letter have significantly different medians.

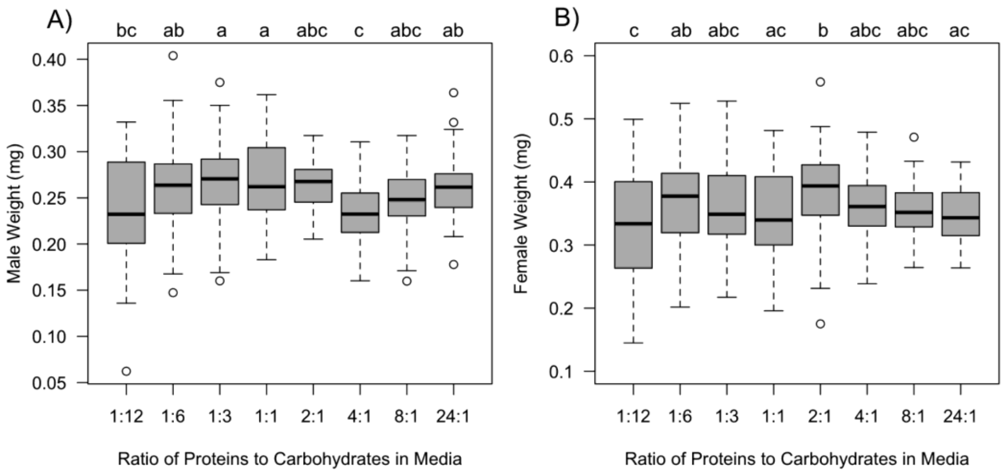

Supplementary Figure S7. Boxplot of weights of adult male (A) and female (B) *Drosophila suzukii* raised on media differing in protein: carbohydrate (P:C) ratio (with antimicrobials). The box encloses values between the first and third quartiles of the data (the inter-quartile range (IQR)), whereas the horizontal bar within the box indicates the median. Whiskers extend from the box to largest/smallest values that are within 1.5 × the IQR of the box. Values outside that range are outliers and are indicated by circles. Boxplots that are not sharing a letter have significantly different means.
